# Review conclusions by Ernst and Canter regarding spinal manipulation refuted

**DOI:** 10.1186/1746-1340-14-14

**Published:** 2006-08-03

**Authors:** Gert Bronfort, Mitchell Haas, David Moher, Lex Bouter, Maurits van Tulder, John Triano, Willem JJ Assendelft, Roni Evans, Simon Dagenais, Anthony Rosner

**Affiliations:** 1 Northwestern Health Sciences University, 2501 W 84^th ^St, Bloomington, MN 55431, USA; 2 Western States Chiropractic College, 2900 NE 132^nd ^Ave, Portland OR 97230, USA; 3 Chalmers Research Group, Evidence-based Practice Center, Departments of Pediatrics, Epidemiology and Community Medicine, University of Ottawa, 401 Smyth, Ottawa ON, K1H8L1, Canada; 4 Institute for Research in Extramural Medicine, Vrije Universiteit Medical Centre, The Netherlands; 5 Texas Back Institute, 6300 W. Parker Road, Plano Texas 75093, USA; 6 Department of General Practice and Nursing Home Medicine, LUMC Medical Centre, Leiden, The Netherlands; 7 Foundation for Chiropractic Education and Research, 1330 Beacon St #315, Brookline MA 02446, USA

## Abstract

In the April 2006 issue of the Journal of Royal Society of Medicine, Ernst and Canter authored a review of the most recent systematic reviews on the effectiveness of spinal manipulation for any condition. The authors concluded that, except for back pain, spinal manipulation is not an effective intervention for any condition and, because of potential side effects, cannot be recommended for use at all in clinical practice. Based on a critical appraisal of their review, the authors of this commentary seriously challenge the conclusions by Ernst and Canter, who did not adhere to standard systematic review methodology, thus threatening the validity of their conclusions. There was no systematic assessment of the literature pertaining to the hazards of manipulation, including comparison to other therapies. Hence, their claim that the risks of manipulation outweigh the benefits, and thus spinal manipulation cannot be recommended as treatment for any condition, was not supported by the data analyzed. Their conclusions are misleading and not based on evidence that allow discrediting of a large body of professionals using spinal manipulation.

## Background

In the April 2006 issue of the Journal of Royal Society of Medicine, Ernst and Canter authored a review of the most recent published systematic reviews on the effectiveness of spinal manipulation for any condition, including back pain, neck pain, and headache [1]. The authors concluded that data from the systematic reviews did not demonstrate spinal manipulation to be an effective intervention for any condition with the exception of back pain, where it was superior to sham manipulation but not better than conventional treatments. They also stated that manipulation cannot be recommended for use in clinical practice because of the potential side effects. The purpose of this commentary is to provide a critical appraisal of their review based on standard systematic review methodology [2].

## Discussion

The Ernst and Canter review is an example of some of the pitfalls associated with conducting reviews that do not adhere to standard systematic review methodology, thus threatening the validity of the conclusions. The authors used a broad sweeping approach to conduct their review that appears to have resulted in misinterpretation of some of the evidence. This led to misleading conclusions regarding the value of spinal manipulation.

First, the authors chose to only summarize reviews published after 2000 without providing a rationale or assessing the impact of this censored, truncated approach. Based on the inclusion and exclusion criteria, the review excluded at least three eligible reviews [3-5] and included at least one review that we do not consider systematic [6]. The review did not reference the eight excluded studies to enable readers to verify the judgments made.

Second, the authors elected to assess the quality of included reviews quite loosely even though more robust and clinimetrically sound approaches are available and have been widely used by others [7]. The authors only made casual comments about certain reviews being more important than others. Such an approach is prone to bias and unnecessary subjectivity [8].

Third, the authors did not report on any pre-specified rules to evaluate the evidence in aggregate and did not perform any sensitivity analysis to test the robustness of their conclusions. Inference about the overall evidence supporting or refuting spinal manipulation was solely based on extraction of text from the conclusions of the individual reviews. The methodological quality and validity of the included reviews apparently were not assessed. There was at least one example of the extracted information from one of their own review abstracts which was in conflict with their reported results [9].

Fourth, there was no attempt made to analyze the nature of discordance between the selected reviews' conclusions for each clinical condition. In our view, this should have included consideration of the study question, methodology and quality of the reviews, as well as the number of randomized trials included in each review. The authors claim that they authored or coauthored 3 of the 16 included reviews and that these all were unbiased and of high quality. From their own table and reference list it is evident that 5 of the 16 reviews (all negative conclusions) were authored or coauthored by Ernst [9-13]. As to the methodological quality of these reviews, we leave it to the scientific community to judge.

Ernst and Canter referred to a study by them which concluded that there was a strong association between positive findings and authorship by chiropractors [14]. However, this study did not include any systematic review assessed in their current review of reviews. Furthermore, the assertion that the overly positive reviews were authored by the same chiropractor is somewhat misleading, as these reviews [15,16] had multi-disciplinary authorship. We wonder why Ernst and Canter, in the interest of being unbiased, did not entertain the possibility that reviews which had no authors with expertise in spinal manipulation were biased. It is very well possible that having content expertise onboard is needed for drawing clinically sensible conclusions.

Additionally, we challenge the implicit assumption used by Ernst and Canter to reach the conclusion that certain systematic reviews show that spinal manipulation is not effective. This assumes that manipulation must outperform other treatments to be considered effective or appropriate care. An example of this is their interpretation, "no proof of effectiveness of spinal manipulation," of the most recent Cochrane review by Assendelft et al [17], which concluded that manipulation was superior to sham/placebo but not better than other types of therapy for low back pain. However, not being superior to other types of therapy does not mean that manipulation is not effective, a fact acknowledged by Assendelft et al in their review [17]. Consistent with that, a very recent systematic review of Cochrane reviews concluded that spinal manipulation is an effective treatment option for low back pain [18].

Ernst and Canter did not conduct or cite a systematic review of the hazards of manipulation including comparison to other therapies. Hence, the claim that the risks of manipulation outweigh the benefits, and thus spinal manipulation cannot be recommended as treatment for any condition, was not supported by the data analyzed.

## Conclusion

The conclusions by Ernst and Canter were definitely not based on an acceptable quality review of systematic reviews and should be interpreted very critically by the scientific community, clinicians, patients, and health policy makers. Their conclusions are certainly not valid enough to discredit the large body of professionals utilizing spinal manipulation.

## Competing interests

The author(s) declare that they have no competing interests.

## Authors' contributions

All authors critically appraised the Ernst and Canter review. G. Bronfort wrote the first draft of the commentary. The remaining authors provided suggestions for changes to the draft. These were all incorporated and the final draft was approved by all authors.
